# Role of 5HT1A Receptors in the Neuroprotective and Behavioral Effects of Cannabidiol in Hypoxic–Ischemic Newborn Piglets

**DOI:** 10.3389/fphar.2022.925740

**Published:** 2022-07-18

**Authors:** Lorena Barata, María de Hoz-Rivera, Angela Romero, María Martínez, Laura Silva, María Villa, Leticia Campa, Laura Jiménez-Sánchez, José Martínez-Orgado

**Affiliations:** ^1^ Servicio de Neonatología, Hospital Clínico San Carlos, IdISSC, Madrid, Spain; ^2^ Fundación para La Investigación Biomédica del Hospital Clínico San Carlos (IdISSC), Madrid, Spain; ^3^ Centro de Investigación Biomédica en Red de Salud Mental (CIBERSAM), Spanish National Research Council (CSIC), Institut d'Investigacions Biomèdiques de Barcelona (IIBB), Institut d'Investigacions August Pi i Sunyer (IDIBAPS), ISCIII, Madrid, Spain; ^4^ Centro de Investigación Biomédica, Instituto de Biotecnoloía, Universidad de Granada, Granada, Spain; ^5^ Instituto de Investigación Biosanitaria de, Granada, Spain

**Keywords:** hypoxia-ischemia, cannabidiol, serotonin receptors, neuroprotection, mood disorders, piglets

## Abstract

**Background:** Hypoxic–ischemic (HI) insults have important deleterious consequences in newborns, including short-term morbidity with neuromotor and cognitive disturbances. Cannabidiol (CBD) has demonstrated robust neuroprotective effects and shows anxiolytic/antidepressant effects as well. These effects are thought to be related to serotonin 5-HT_1A_ receptor (5HT_1A_R) activation. We hereby aimed to study the role of 5HT_1A_R in the neuroprotective and behavioral effects of CBD in HI newborn piglets.

**Methods:** 1-day-old piglets submitted to 30 min of hypoxia (FiO2 10%) and bilateral carotid occlusion were then treated daily with vehicle, CBD 1 mg/kg, or CBD with the 5HT_1A_R antagonist WAY 100635 1 mg/kg 72 h post-HI piglets were studied using amplitude-integrated EEG to detect seizures and a neurobehavioral test to detect neuromotor impairments. In addition, behavioral performance including social interaction, playful activity, hyperlocomotion, and motionless periods was assessed. Then, brain damage was assessed using histology (Nissl and TUNEL staining) and biochemistry (proton magnetic resonance spectroscopy studies.

**Results:** HI led to brain damage as assessed by histologic and biochemistry studies, associated with neuromotor impairment and increased seizures. These effects were not observed in HI piglets treated with CBD. These beneficial effects of CBD were not reversed by the 5HT_1A_R antagonist, which is in contrast with previous studies demonstrating that 5HT_1A_R antagonists eliminated CBD neuroprotection as assessed 6 h after HI in piglets. HI led to mood disturbances, with decreased social interaction and playfulness and increased hyperlocomotion. Mood disturbances were not observed in piglets treated with CBD, but in this case, coadministration of the 5HT_1A_R antagonist eliminates the beneficial effects of CBD.

**Conclusion:** CBD prevented HI-induced mood disturbances in newborn piglets by acting on 5HT_1A_R. However, 5HT_1A_R activation seems to be necessary for CBD neuroprotection only in the first hours after HI.

## 1 Introduction

Hypoxic–ischemic (HI) brain damage affects 1–3/1000 live-term newborns, representing one of the leading causes of death and permanent disability in infants (Zhou et al., 2020). The clinical picture of newborns with severe HI encephalopathy is that of deep coma, but newborns with mild-to-moderate HI encephalopathic show a varied neurologic picture that includes increased or decreased muscle tone and reflexes, fluctuating alertness, jitteriness, hyperexcitability, and seizures, depending on the timing and severity of the insult (Sarnat and Sarnat, 1976; Thompson et al., 1997). This situation makes the HI newborn particularly susceptible to the stress inherent in the application of the current standard of care and therapeutic hypothermia (Zhou et al., 2020), which determines that HI newborns under hypothermia very often receive sedatives as opioids (Berube et al., 2020). Since hypothermia affects the metabolism of opiates, opiate treatment during hypothermia easily causes unwanted side effects such as cardiorespiratory instability and neurologic depression (Berube et al., 2020).

Since hypothermia does not benefit a substantial number of HI newborns, additional or alternative neuroprotective strategies are being investigated (Zhou et al., 2020). Cannabidiol, the noneuphoric component of *Cannabis sativa*, has demonstrated robust neuroprotective effects in different models of HI brain damage in newborns, modulating inflammation, excitotoxicity, and oxidative stress, thus protecting neurons and glial cells (Martínez-Orgado et al., 2021). Although the mechanisms of action of CBD are not fully understood, it is known that CBD acts on different signaling pathways by activating different receptors such as the peroxisome proliferator-activated receptor gamma (PPARγ), G protein-coupled receptor 55 (GPR5), transient receptor potential vanilloid subtype 1 (TRPV1), and serotonin (5-hydroxytryptamine, 5-HT) 5-HT_1A_ receptors (de Almeida and Devi, 2020; Silvestro et al., 2020; Martínez-Orgado et al., 2021; Melas et al., 2021). In fact, the main neuroprotective effects of CBD are believed to be related to the activation of the 5-HT_1A_ receptor (5HT_1A_R) (Silvestro et al., 2020). This is interesting since CBD exerts modulatory effects on mood disturbances such as anxiety, panic, or depression mainly through the activation of 5HT_1A_R (De Gregorio et al., 2019; de Almeida and Devi, 2020; Melas et al., 2021). There are no reports on the effects of CBD on mood disturbances in newborn animals. We have reported that 5HT_1A_R blockade abolishes the neuroprotective effects of CBD in HI piglets as assessed 6 h after the insult, but in that study, piglets remained sedated, which together with the short follow-up time made it impossible to assess the effects of CBD and 5HT1AR blockers in the piglet behavior (Pazos et al., 2013).

The aim of this work was to study the involvement of 5HT1AR receptors on CBD neuroprotection and the effects of CBD in HI-induced mood disorders. For this, we used a model with high translational value, in which neuromotor and behavioral evaluations are carried out in HI piglets in addition to histological and biochemical studies (Lafuente et al., 2011; Barata et al., 2019b).

## 2 Methods

### 2.1 Experimental Model

All procedures complied with European Directive (2010/63/EU) and Spanish (RD 53/2013) regulations for the protection of experimental animals and were approved by the Animal Welfare Ethics Committee of Hospital Clinico San Carlos in Madrid, Spain (ProEx 175/14). All experimental procedures were designed and carried out by personnel qualified in Laboratory Animal Science, following FELASA recommendations in categories B and C to reduce animal stress and enhance animal welfare. All surgery was performed under adequate anesthesia and analgesia, and great effort was made to minimize suffering and reduce the number of animals used. Furthermore, all experimental procedures on animal welfare (anesthesia and analgesia; drug and substance administration) and euthanasia of the animals were conducted in compliance with FELASA recommendations. The sample size was calculated accordingly and based on previous results experiments using the same model (Lafuente et al., 2011; Barata et al., 2019b).

#### 2.1.1 Induction of Hypoxia–Ischemia

The protocol was based on the model reported extensively elsewhere (Lafuente et al., 2011; Barata et al., 2019b). In short, one-day-old male Landrace-White large piglets provided by a local certified farm the day the experiment started were intubated and then mechanically ventilated (Babylog8000, Dräger, Germany) under sevoflurane anesthesia (5% induction and 1% maintenance) and received morphine chloride 1 0.1 mg/kg I.M. A right jugular vein indwelling catheter was inserted and kept in place over the entire experimental period for i.v. drug administration. Nontraumatic stainless-steel wires were placed into the piglet head’s scalp to continuously monitor brain activity by amplitude-integrated electroencephalography (aEEG; BRM3, BrainZ Instruments, Auckland, New Zealand). Body temperature was maintained at 37.5–38.0°C by an air-warmed blanket. Piglets were then randomly assigned to the experimental group in a blind fashion. In the HI groups, each carotid artery was exposed and surrounded by an elastic band. Then, HI piglets underwent a 20-minute–long cerebral HI insult by interrupting carotid blood flow by pulling out the carotid bands and reducing inspired oxygen fraction (FiO_2_) to 10%. The 20-min countdown started when aEEG trace became flat. At the end of the HI period, the carotid flow was restored and FiO_2_ increased to 21%. Piglets were similarly managed but without HI insult and were used as reference (sham group, SHM, *n* = 6).

#### 2.1.2 Drug Treatment

Thirty minutes after HI piglets were randomly assigned to receive i.v. vehicle (HV, *n* = 16) or CBD (Abcam, Cambridge, UK) 1 mg/k (HC, *n* = 15), alone or with the antagonist of serotonin 5HT_1A_R WAY100635 (Tocris Bioscience, Abingdon, UK) 1 mg/kg (HCW, *n* = 9). CBD was prepared in a 5 mg/ml formulation of ethanol : colliphor : saline at a ratio of 2 : 1 : 17 and further diluted in saline to administer a total volume of 10 ml by infusion pump over 10 min. WAY 100635 was administered 15 min before CBD and dissolved in the same vehicle. Doses of vehicle, CBD, and WAY 100635 were repeated every 24 h, three doses in total. Doses were selected following previous *in vivo* experiments by our group in HI piglets (Pazos et al., 2013; Barata et al., 2019a).

#### 2.1.3 Follow-Up

Then, after fully regaining consciousness from the experimental procedure and anesthesia, piglets were extubated and transferred to the animal care facility. Piglets were housed in stainless steel cages with 0.5 m^2^ per animal in a well-controlled warm-temperature environment. The cage contained a pending rope and a plastic ball for environmental enrichment. From 24 h post-insult, piglets were fed with artificial piglet formula (Nutrilac Milk Replacer for young piglets, Joosten Products B.V. Weert, Netherlands) every 3-4 h by a catheter attached to the examiner’s finger to assess suckling. The examiner was blinded to the experimental group. Ceftazidime 15 mg/kg i.v. was administered every 12 h to prevent infections and acetaminophen 15 mg/kg i.v. was administered every 8 h for pain control. Seventy-two hours after the HI insult, piglets were sacrificed by KCl infusion under anesthesia (5% sevoflurane). Both carotid arteries were cannulated to perfuse brains with heparin in cold saline until clean fluid flew from the sectioned jugular arteries. Then, the brains were removed from the skull and sectioned. Brain slices from the left hemisphere were placed into 4% paraformaldehyde for histologic analysis, whereas those from the right hemisphere were snap frozen in isopentane and stored at −80°C for spectroscopy studies.

### 2.2 Neurobehavioral Assessment

Each morning from 8 to 10 a.m., the piglets were studied in the room where their cage was placed and video-recorded by one examiner. All video recordings were subsequently evaluated and scored when appropriate by three researchers blinded to the experimental group to obtain a mean value of the different items. A neurobehavioral score (NBS: 8–36 pts) (Lafuente et al., 2011; Barata et al., 2019b), based on that by LeBlanc et al. (LeBlanc et al., 1991), was carried out and video-recorded every 24 h, measuring alertness, behavior, muscle tone, standing, walking, and eating (Table 1).

**TABLE 1:** Neurobehavioral score (NBS).

*Mental status*	Coma	0
	Stupor	1
	Lethargy	3
	Awake	4
*Behavior*	None	0
	Weak	1
	Aggressive	3
	Normal	4
*Pupils*	Nonreactive	1
	Slow, asymmetric	2
	Normal	3
*Vestibulo–ocular reflex*	Absent Nystagmus	1
	Normal	2
		3
*Stepping* (*barrow*)	None	1
	Just fore/hind paws	2
	Normal	3
*Righting*	Absent/present	1
		2
*Muscle tone*	Atonic/hypertonic	1
	Partially atonic	2
	Partially hypertonic	3
	Normal	4
*Standing*	No	1
	Paresis	2
	Unsteady	3
	Normal	4
*Walking*	No	1
	Paresis	2
	Falling	3
	Normal	4
*Eating*	No suckling reflex	1
	Weak reflex, tube feeding	2
	No appetite	3
	Brief suckling	4
	Normal	5

Piglets were also video recorded during free wandering for 5 min. According to previous studies (Barata et al., 2019b), time spent interacting with caretakers (following caretaker’s movements, pushing, nosing, sniffing, and licking) was considered social interaction time, whereas time spent in playful behavior (pushing, nosing, sniffing, and biting) with some known object (the sheet used for feeding) considered playfulness time. Time spent in unpurposive quick movements was considered hyperactivity time. Time with no activity while standing and lasting more than 5 s was considered motionless time. After the neurobehavioral assessment, piglets were wrapped up with a blanket and held by an examiner who sat in front of the aEEG device, to assess cerebral activity for over 10 min. Test start time and time when the piglet stopped fighting against immobilization (FAI) were recorded. The time of restlessness during slight restraint for aEEG performance was considered an indicator of piglet anxiety.

### 2.3 Seizures

Raw EEG traces were manually reviewed for electric seizures (periods of rhythmic activity starting with a sudden increase in voltage of at least 2 μV, accompanied by a narrowing of the band of aEEG activity, lasting at least 15 s).

### 2.4 Histologic Analysis

Histological studies were performed in 4 µm thick coronal sections obtained from fixed brain hemispheres, as reported previously (Lafuente et al., 2011; Pazos et al., 2013; Barata et al., 2019b). Areas of 1 mm^2^ in the central three lobes of the parietal cortex and the adjacent CA1 area of the hippocampus were examined by two skilled investigators blinded to the experimental group. Neuronal necrosis was identified by Nissl staining, according to previous studies (Lafuente et al., 2011; Pazos et al., 2013; Barata et al., 2019b). Cell death was further studied in the cortex by TUNEL staining (DeadEnd Colorimetric TUNEL System, Promega, Spain) as reported elsewhere (Lafuente et al., 2011; Pazos et al., 2013; Barata et al., 2019b). Samples were visualized and photographed with a confocal TCS SP5 confocal microscope (Leica Microsystems, Wetzlar, Germany).

### 2.5 Proton Magnetic Resonance Spectroscopy (^1^H-NMR)

*Ex vivo* ^1^H spectrum was performed on a Bruker Avance 11.7 T spectrometer (Bruker BioSpin, Karlsruhe, Germany) equipped with a 4 mm triple channel 1H/13C/31P HR-MAS (High-Resolution Magic Angle Spinning) resonance probe. HMRS was performed in the MRI Unit of Instituto Investigaciones biomédicas “Alberto Sols” (CSIC-UAM, Madrid, Spain). Frozen cortex samples (5-10 mg weight) were introduced into a 50 µL zirconia rotor (4 mm OD) with 50 µL D2O and spun at 5000 Hz at 4°C to prevent tissue degradation processes. Two types of monodimensional proton spectra were acquired using water suppressed spin echo Carr Purcell Meiboom Gill (CPMG) sequence with 36 and 144 ms echo time and 128 scans. Data were collected into 64k data points using a spectral width of 10 kHz (20ppm) and water presaturation during a relaxation delay of 2 s, total acquisition of 16 min. All spectra were analyzed using the LC Model software; only peak concentrations obtained with a standard deviation lower than 20% were accepted. The content of glutamate (Glu), lactate (Lac), and N-acetylaspartate (NAA) was normalized to the creatine content.

### 2.6 Brain Neurotransmitter Concentration

Brain concentration of norepinephrine (NE), dopamine (DA), and serotonin (5-HT) was measured in homogenate from frozen brain tissue by high-performance liquid chromatography (Barata et al., 2019b). In short, tissues were ultrasonically homogenized in 0.4 M perchloric acid with 5 mM sodium metabisulfite, 8.3 uM cysteine, and 0.3 mM of EDTA. Samples were then centrifuged at 12,000 g for 30 min at 4°C. The three neurotransmitters were separated using an Ultrasphere 3 µm column (7.5 cm × 0.46 cm, Beckman, San Ramon, CA) and detected with a Hewlett–Packard amperometric detector (Palo Alto, CA). The mobile phase comprised 0.15 M KH2PO4, 0.46 mM octyl sodium sulfate, 0.5 Mm EDTA (pH 2.8 adjusted with phosphoric acid), and 12% methanol and was pumped at a rate of 0.6 ml/min.

### 2.7 Statistical Analysis

Data were analyzed with a statistical software package (GraphPad Prism 9.1.0; GraphPad Software, San Diego, CA, United States ). After assessing the normality of data distribution using the Shapiro–Wilk test, data showing normal distribution were displayed as mean ± SEM and compared using one-way ANOVA with Holm–Šidák’s test for multiple comparisons. Data showing a nonnormal distribution were displayed as median (CI 95%) and compared using Kruskall–Wallis with Dunn`s test for multiple comparisons. The distribution of qualitative parameters was studied using the *X* ^2^ test. *p* < 0.05 was considered significant.

## 3 Results

### 3.1 General Data

Piglets from all groups were similar in weight at the moment of the study (1.70 ± 0.1, 1.73 ± 0.1, 1.69 ± 0.1, and 1.78 ± 0.1 kg for SHM, HV, HC, and HCW, respectively, *p* = 0.83). Four out of 16 HV, three out of 15 HC, and one out of nine HCW piglets died in the first 24 h after HI (X^2^ = 0.69, *p* = 0.7). This represents a mortality of 27.5%, similar to that reported (Lafuente et al., 2011; Barata et al., 2019b). None of the SHM piglets died during the follow-up.

WAY100635 exerted no demonstrable effects on SHM or vehicle-treated HI animals in all studies. Therefore, those piglets were not included in the final data analysis.

### 3.2 Assessment of HI-Induced Brain Damage

**FIGURE 1:**
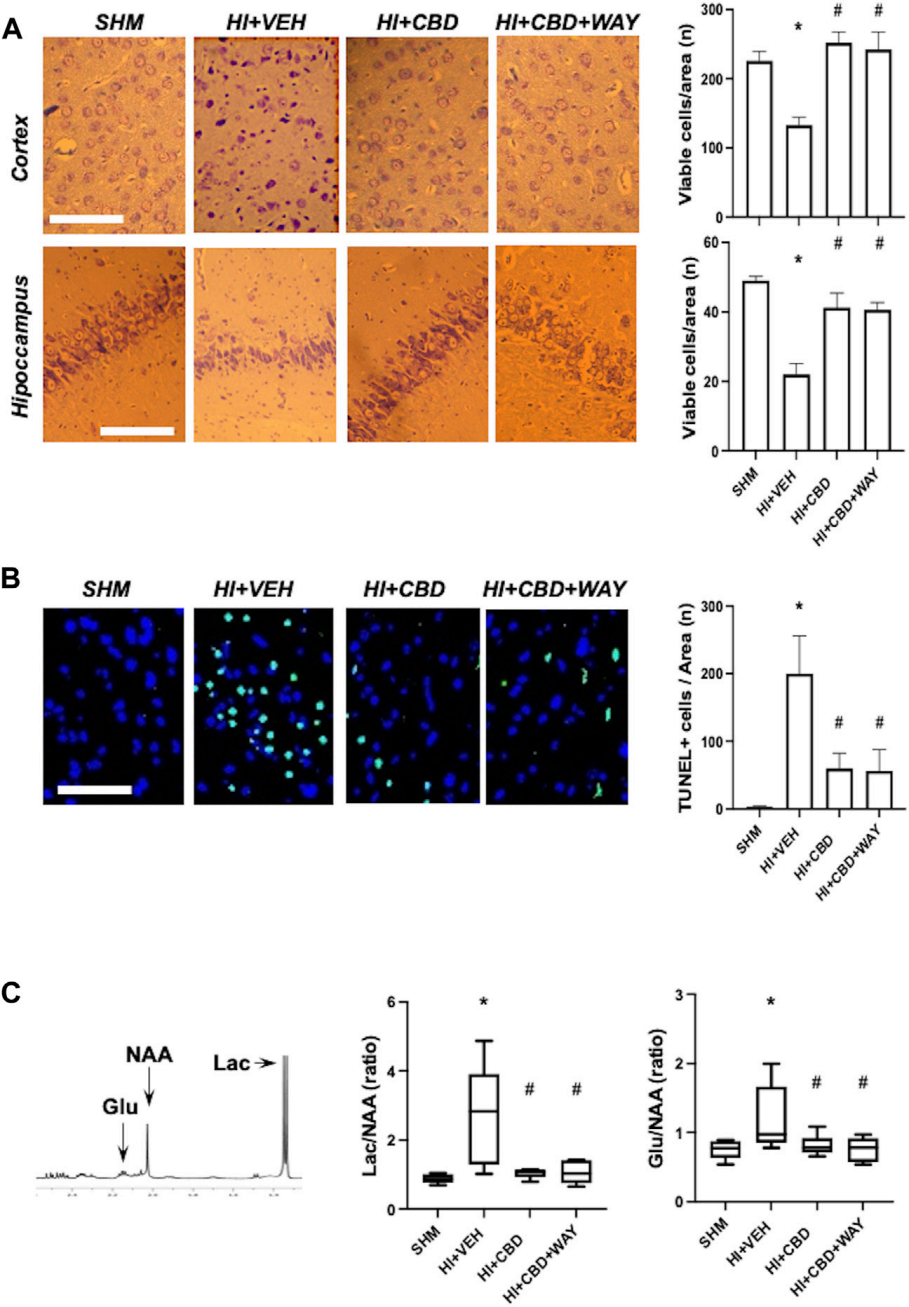
Brain damage assessment 72 h after hypoxic–ischemic insult in brain samples from piglets treated with vehicle (HV), cannabidiol (HC), and WAY 100635 (HCW). Non-hypoxic–ischemic piglets served as controls (SHM). **(A)** Representative microphotographs (*left*) and quantification (*right*) of viable neurons using Nissl staining in the temporoparietal cortex and adjacent CA1 area of the hippocampus. **(B)** Representative microphotographs (*left*) and quantification (*right*) of cell death using TUNEL staining. Bars represent the mean (SEM) of 6–12 experiments. Scale bar: 100 µm. **(C)** Results from proton magnetic resonance spectroscopy. Lac: lactate. Glu: glutamate. NAA: N-acetylaspartate. Boxes represent the median (IQR) and Whiskers' maximum and minimum values. (*) *p* < 0.05 vs. SHM and (#) *p* < 0.05 vs. HV by ANOVA with Holm–Šidack test for multiple comparisons **(A, B)** or Kruskall–Wallis with Dunn’s test for multiple comparisons **(C)**.

HI led to a reduction in the density of viable neurons in the cortex and hippocampus, as assessed by Nissl staining and induced a dramatic increase in the number of TUNEL+ cells (Figure 1A). Such effects of HI were not observed in CBD-treated piglets, which showed viable neuron and TUNEL+ cell density similar to SHM (Figure 1B). Coadministration of the 5HT_1A_R blocker did not modify the protective effects of CBD (Figures 1A, B).

HI insult led to brain metabolic derangement, as reflected by the increase in Lac/NAA ratio in the ^1^H-NMR studies (Figure 1C). In addition, HI led to increased excitotoxicity as reflected by the higher Glu/NAA ratio in HI than in SHM animals (Figure 1C). Lac/NAA and Glu/NAA increase was not observed in HI piglets treated with CBD (Figure 1C). Coadministration of the 5HT_1A_R blocker did not modify the protective effects of CBD (Figure 1C).

### 3.3 Seizures

The aEEG exam revealed electric seizures 72 h after HI in four out of 12 HV piglets but in none of the SHM, HC, or HCW piglets (*X* ^2^ = 8.65, *p* = 0.03). The seizure burden in those piglets was 17.9 ± 6.8 (1.4–32.5% of the recording time).

### 3.4 Functional and Behavioral Studies

HI insult led to a decrease in NBS global score 72 h after HI in HV animals (Table 2). Such a decrease was because of the decrease in motor and behavioral scores and in eating proficiency (Table 2). Such impairments were not observed in HC animals, which showed an NBS global score similar to SHM animals (Table 2). HCW animals showed a trend of worse behavioral and eating performance, but this did not reach statistical significance (Table 2).

**TABLE 2:** Neurobehavioral assessment.

	SHM (n = 6)	HI + VEH (*n* = 12)	HI + CBD (*n* = 12)	HI + CBD + WAY (*n* = 8)
Conscience	4.0 (4.0, 4.0)	4.0 (3.6, 4.1)	4.0 (4.0, 4.0)	4.0 (4.0, 4.0)
Behavior	4.0 (3.6, 4.1)	3.0 (1.7, 3,3)*	4.0 (3.3, 4.1)^#^	3.5 (2.8, 3.9)
Pupils	3.0 (3.0, 3.0)	3.0 (3.0, 3.0)	3.0 (3.0, 3.0)	3.0 (3.0, 3.0)
VOR	3.0 (3.0, 3.0)	3.0 (3.0, 3.0)	3.0 (3.0, 3.0)	3.0 (3.0, 3.0)
Barrow	3.0 (3.0,3 0.0)	3.0 (1.8, 3.1)	3.0 (2.7, 3.0)	3.0 (2.7, 3.0)
Righting	2.0 (2.0, 2.0)	2.0 (1.3, 2.1)	2.0 (2.0, 2.0)	2.0 (2.0, 2.0)
Tone	4.0 (4.0, 4.0)	3.2 (1.9, 3.6)*	4.0 (3.6, 4.0)	4.0 (3.6, 4.0)
Standing	4.0 (4.0, 4.0)	3.7 (2.2, 4.1)	4.0 (3.5, 3.9)	4.0 (3.6, 4.0)
Walking	4.0 (4.0, 4.0)	3.5 (2.2, 4.0)	4.0 (3.6, 4.0)	3.7 (3.5, 3.9)
Eating	5.0 (4.3, 5.3)	4.0 (2.2, 4.4)*	5.0 (4.5, 5.0)^#^	4.0 (3.4, 4.5)
Total	36.0 (35.1, 36.2)	31.5 (23.6, 34.5)*	35.5 (33.8, 35.8)^#^	34.0 (32.7, 34.7)

Median (CI, 95%). SHM: sham. HI**:** hypoxia–ischemia. VEH: vehicle. CBD: cannabidiol. WAY: WAY, 100635. VOR: vestibulo–ocular reflex. (∗) *p* < 0.05 vs. SHM, and (#) *p* < 0.05 vs. HV, by ANOVA, with Holm–Šidack test for multiple comparisons.

**FIGURE 2:**
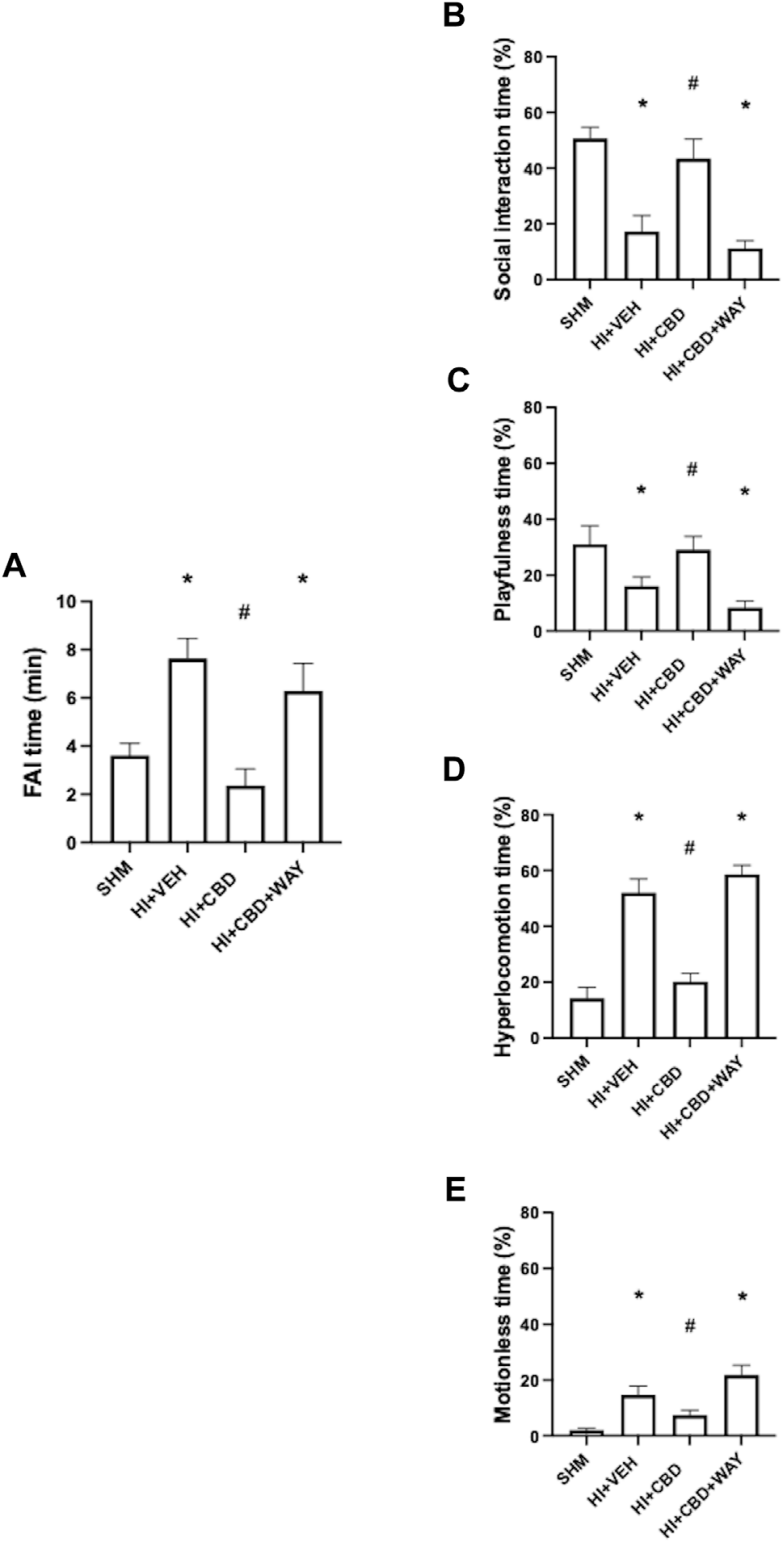
Neurobehavioral assessment 72 h after hypoxic–ischemic insult in piglets treated with vehicle (HV), cannabidiol (HC), and WAY 100635 (HCW). Non-hypoxic–ischemic piglets served as controls (SHM). **(A)** Time spent fighting against immobilization (FAI) (*left*). Relative time (*right*) spent in **(B)** social interaction, **(C)** playfulness, **(D)** hyperlocomotion, and **(E)** motionless periods. Bars represent the mean (SEM) of 6–12 experiments. (∗) *p* < 0.05 vs. SHM and (#) *p* < 0.05 vs. HV by ANOVA with Holm–Šidack test for multiple comparisons.

HI led to mood disturbances in piglets as observed in HV piglets 72 h after HI. HV piglets showed increased anxiety as reflected by a nearly twofold increase in the FAI time (Figure 2A). In HV piglets there was a reduction in social interaction and playful activity (Figures 2B, C). By contrast, HV piglets showed increased hyperactivity time and increased motionless time (Figures 2D, E). Mood disturbances were not observed in HI piglets treated with CBD. Thus, playfulness, social interaction, hyperactivity, and motionless time, and FAI time, were similar in HC and SHM animals (Figures 2A–E). In this case, coadministration of the 5HT_1A_R antagonist abolished the protective effects of CBD, with HCW showing similar responses to those of HV animals (Figures 2A–E)

### 3.5 Monoamine Brain Concentration

**FIGURE 3:**
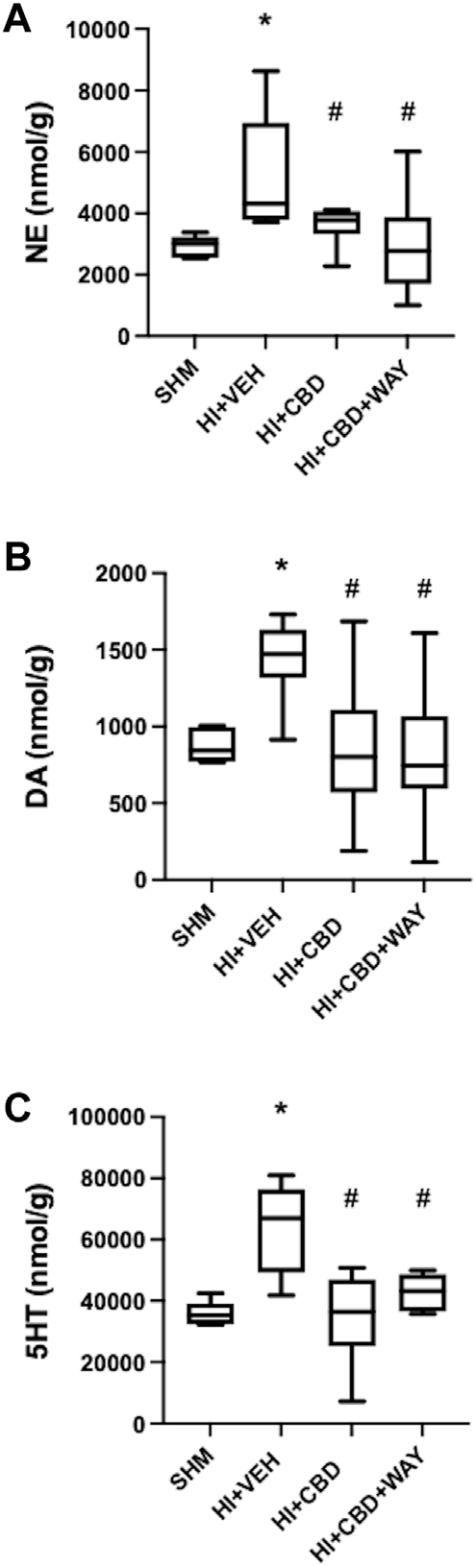
Brain monoamine concentration 72 h after hypoxic–ischemic insult in brain samples from piglets treated with vehicle (HV), cannabidiol (HC), and WAY 100635 (HCW). Non-hypoxic–ischemic piglets served as controls (SHM). Boxes represent the median (IQR) and Whiskers' maximum and minimum values of brain concentration of **(A)** norepinephrine (NE), **(B)** dopamine (A), and **(C)** 5-hydroxytriptamine (5-HT). (∗) *p* < 0.05 vs. SHM and (#) *p* < 0.05 vs. HV by Kruskall–Wallis with Dunn’s test for multiple comparisons.

There was an increase in NE, DA, and 5HT concentration in brain samples obtained from HV animals 72 h after HI (Figures 3A–C). Such an increase was prevented by CBD administration, with HC animals showing similar monoamine brain concentration similar to SHM (Figures 3A–C). Coadministration of the 5HT_1A_R blocker did not modify the protective effects of CBD (Figures 3A–C).

## 4 Discussion

HI insult led to brain damage in newborn piglets as assessed 72 h after the insult. HI led to the reduction in the cortex of viable neurons in Nissl-stained preparations and increased TUNEL positive cells, increased Lac/NAA ratio in ^1^H-NMR studies, increased seizures, and the development of neuromotor deficits, as has been described (Barata et al., 2019b). All these features were not observed in piglets treated with CBD at a daily dose of 1 mg/kg for 3 days. The beneficial histological and neuromotor effects of CBD evaluated 72 h after HI insults in piglets have also been described after administering a single dose of CBD (Lafuente et al., 2011).

### 4.1 5HT_1A_R Antagonism Did Not Affect CBD Neuroprotective Effects

In this work, the neuroprotective effects of CBD evaluated 72 h after HI were not affected by the coadministration of 5HT_1A_R antagonist. However, we have reported that coadministration of the same 5HT_1A_R antagonist results in loss of CBD neuroprotection assessed 6 h after a HI insult in piglets (Pazos et al., 2013). CBD is a well-known allosteric agonist of 5HT_1A_R (Russo et al., 2005; de Almeida and Devi, 2020; Silvestro et al., 2020; Melas et al., 2021). It is generally accepted that the main neuroprotective effects of CBD are related to 5HT_1A_R activation (Silvestro et al., 2020). Our results suggest that this is the case for CBD neuroprotection in the brain of newborn piglets in the first hours after HI, but later, CBD could exert neuroprotective actions by mechanisms independent of 5HT_1A_R activation. Although other mechanisms were not studied in this work, it has been widely described that CBD can induce neuroprotective effects by activating other receptors such as PPARγ, GPR55, and TRPV1, increasing brain neurotrophic factor (BDNF) expression, modulating adenosine activity, or exerting antioxidant actions related to its molecular structure (Mori et al., 2017; de Almeida and Devi, 2020; Silvestro et al., 2020; Martínez-Orgado et al., 2021; Melas et al., 2021). 5HT_1A_R agonists are neuroprotective in different preclinical models of HI brain damage in adult animals (Aguiar et al., 2021). It is unlikely that the signaling cascade triggered by 5HT_1A_R activation was operational 6 h but not 72 h after HI insult in piglet brains. Indeed, 5HT_1A_R activation was involved in the neurobehavioral effects of CBD in piglets 72 h after HI, as discussed below, suggesting that 5HT_1A_R were operational at that time. Due to this finding, it is also unlikely that 5HT_1A_R activation was less relevant in CBD neuroprotection due to a dramatic reduction of 5HT_1A_R density. A mild reduction of 5HT_1A_R density in the striatum, related to greater destruction of serotoninergic neurons, is described 7 days after HI insults in P3 rats, which present a brain developmental status similar to that of premature infants (Buller et al., 2012). In contrast, the 5HT_1A_R expression is upregulated in P7 rats, which present a brain developmental status similar to that of term infants, 7 days after HI insult (Franco et al., 2019). In addition, the reduction of 5HT_1A_R in P3 rats is associated with a decrease in the brain concentration of 5HT (Buller et al., 2012), an effect not observed in our experiments. However, since we did not directly study either 5HT_1A_R-related signaling pathways or 5HT_1A_R density in the brain, these considerations remain speculative and deserve further study. In a preclinical model of stroke in adult mice, CBD neuroprotection is related to increased regional and intralesional cerebral blood flow, an effect that is dependent on 5HT_1A_R activation (Mishima et al., 2005). It is conceivable that such an effect on cerebral blood flow would be more important in the first few hours after HI, when cerebral blood flow is dramatically reduced (Hassell et al., 2015); however, the relevance of this effect would be much less once brain hypoperfusion evolves into hyperperfusion and then to the normalization of cerebral blood flow (Hassell et al., 2015). More studies are needed, however, to demonstrate that the lack of effect of 5HT_1A_R antagonism on CBD neuroprotection would also be observable in brain areas not explored in our work such as the striatum, cerebellum, or white matter.

### 4.2 HI Insult Led to Mood Disturbances in Piglets

The HI insult also caused behavioral disturbances in the piglets. Although the NBS includes the item “behavior,” its qualitative nature makes it subjective and highly dependent on the examiner’s skills. The additional behavioral assessment that we performed, based on some basic behavioral characteristics of piglets such as playfulness, social interaction, and anxiety responses to immobilization, obtains quantitative and objective parameters (Barata et al., 2019b). HI led to mood disturbances in piglets, as evidenced by reduced social interaction and playtime with objects, suggestive of less attention and/or motivation, and by hyperactivity alternating with motionless episodes. This picture was associated with a greater anxious response during restraint. These features, which are related to brain damage in HI piglets (Barata et al., 2019b), mimic some presence in human newborns two-to-three days after mild-to-moderate HI brain insults, such as jitteriness and attention deficits (Sarnat and Sarnat, 1976; Thompson et al., 1997). We found that mood disturbances coincided with increased brain levels of monoamines such as NE, DA, and 5HT. Increased brain concentration of NE, DA, and 5HT is observed in the brain of adult Wistar rats that develop hypertonia after transient global brain ischemia and is attributed to increased release due to cell destruction along with reduced reuptake due to energy failure (Globus et al., 1992). In piglets, there is a transient increase in brain concentration of the DA transporter in the first days after HI, which is thought to correspond with increased DA levels, related to the damage of dopaminergic neurons in the striatum (Zhang et al., 2012). In our work, hyperlocomotion and anxious response to stress could be attributed to increased DA concentration in the brain (Liu et al., 2018), but motionless and reduced playfulness are more suggestive of depression-like behavior (Kanitz et al., 2004) that is not an effect of increased monoamine concentration (Liu et al., 2018). These features suggest that mood disturbances observed in HI piglets were due to a dysregulation of monoaminergic networks due to brain damage rather than to a direct effect of increased monoamine concentration in the brain. In addition, depressive behavior is linked to reduced BDNF levels in mice brains after acute ischemic insults (Mori et al., 2017), although this was not explored in our work.

### 4.3 5HT_1A_R Antagonism Modified CBD Effects on Mood Disturbances After HI

CBD treatment prevented the development of mood disturbances in piglets after HI. In this case, coadministration of the 5HT_1A_R antagonist eliminated the protective effect of CBD. CBD is a well-known anxiolytic substance (de Almeida and Devi, 2020; Melas et al., 2021) that also shows antidepressant effects (Abame et al., 2021). These effects are mediated by 5HT_1A_R (De Gregorio et al., 2019; de Almeida and Devi, 2020). At 100 mg/kg, CBD can increase 5HT concentration in rat brain (Abame et al., 2021), but at 5 mg/kg, the antipanic effect of CBD is reversed by 5HT_1A_R antagonists, and it is not associated with changes in the concentration of 5HT (Campos et al., 2013). In our case, the CBD dose was even lower than that. Furthermore, the concentration of 5HT in the brain of CBD-treated HI piglets was similar to that of SHM animals, whereas the effects of CBD on mood disturbances disappeared after 5HT_1A_R blockade. The modulatory effects of CBD on brain monoamine concentration after HI were not reversed by 5HT_1A_R blockage. A similar effect on glutamate release was observed, with an increase in Glu/NAA ratio as assessed by ^1^H-NMR studies in HV but not in HC or HCW animals. These data suggest that the modulatory effects of CBD on neurotransmitter release were not due to a direct effect on 5HT_1A_R. CBD is known to reduce the release of neurotransmitter (de Almeida and Devi, 2020; Silvestro et al., 2020; Martínez-Orgado et al., 2021; Melas et al., 2021). But in our case, it could also be the result of the general neuroprotective effects of CBD. Furthermore, CBD normalizes BDNF expression in the mouse brain after hypoxic–ischemic insults, an effect that explains the antidepressant effects of CBD in this condition (Mori et al., 2017).

Taken together, these results support the idea that the effect of CBD on mood disturbances in HI piglets was elicited by direct activation of 5HT_1A_R. Interestingly, in our study CBD prevented the appearance of mood disturbances at a dose of 1 mg/kg. In preclinical studies in adult rodents, anxiolytic or antidepressant effects of acute CBD administration are generally obtained at doses of 2.5 mg/kg or higher, although anxiolytic effects as observed in social interaction tests have been reported for CBD 1 mg/kg in mice (Melas et al., 2021). The robust effects we observed with such a low dose of CBD suggest that an immature brain would be particularly sensitive to 5HT_1A_R agonism by CBD. We have described in the brain of immature rats that HI insults lead to increased expression and signaling of 5HT_1A_R in the first days after HI, an effect of greater magnitude in newborns than in adult rats (Franco et al., 2019).

In conclusion, HI insult led to brain damage in newborn piglets, as observed in histological and biochemistry studies 72 h after HI. At that time, brain damage was associated with increased seizures, and neuromotor deficits, and behavioral disorders, including mood disturbances such as reduced social interaction and playfulness and increased anxiety and hyperactivity. Postinsult CBD administration prevented HI-induced brain damage, and neuromotor deficits and behavioral disturbances. Coadministration of the 5HT_1A_R antagonist WAY 100635 did not modify the beneficial effects of CBD on brain damage and neuromotor performance. This is in contrast to previous studies demonstrating that 5HT1AR blockade abolishes CBD neuroprotection assessed 6 h after HI, suggesting that 5HT1AR activation plays a critical role in CBD neuroprotection only in the first moments after HI. Further studies to determine more precisely the time point after which 5HT1AR antagonism no longer reverses the protective effects of CBD are warranted. In contrast, 5HT1AR blockade eliminated the beneficial effects of CBD on HI-induced mood disturbances, suggesting that these effects were related to 5HT1AR activation. These results indicate that, in addition to its robust neuroprotective effects, CBD could be an interesting candidate to be included in the treatment of HI newborns to mitigate the consequences of stress derived from brain damage and hypothermia treatment.

## Data Availability

The raw data supporting the conclusion of this article will be made available by the authors, without undue reservation, upon request to the corresponding author.
