# Supplementary material for: Role of 5HT1A Receptors in the Neuroprotective and Behavioral Effects of Cannabidiol in Hypoxic–Ischemic Newborn Piglets
Source: Front Pharmacol. 2022 Jul 18;13:925740. doi: 10.3389/fphar.2022.925740 (PMC9341521; doi:10.3389/fphar.2022.925740)
Supplement: Supplementary file 1 [file DataSheet1.PDF]

SUPPLEMENTARY MATERIAL

METHODS

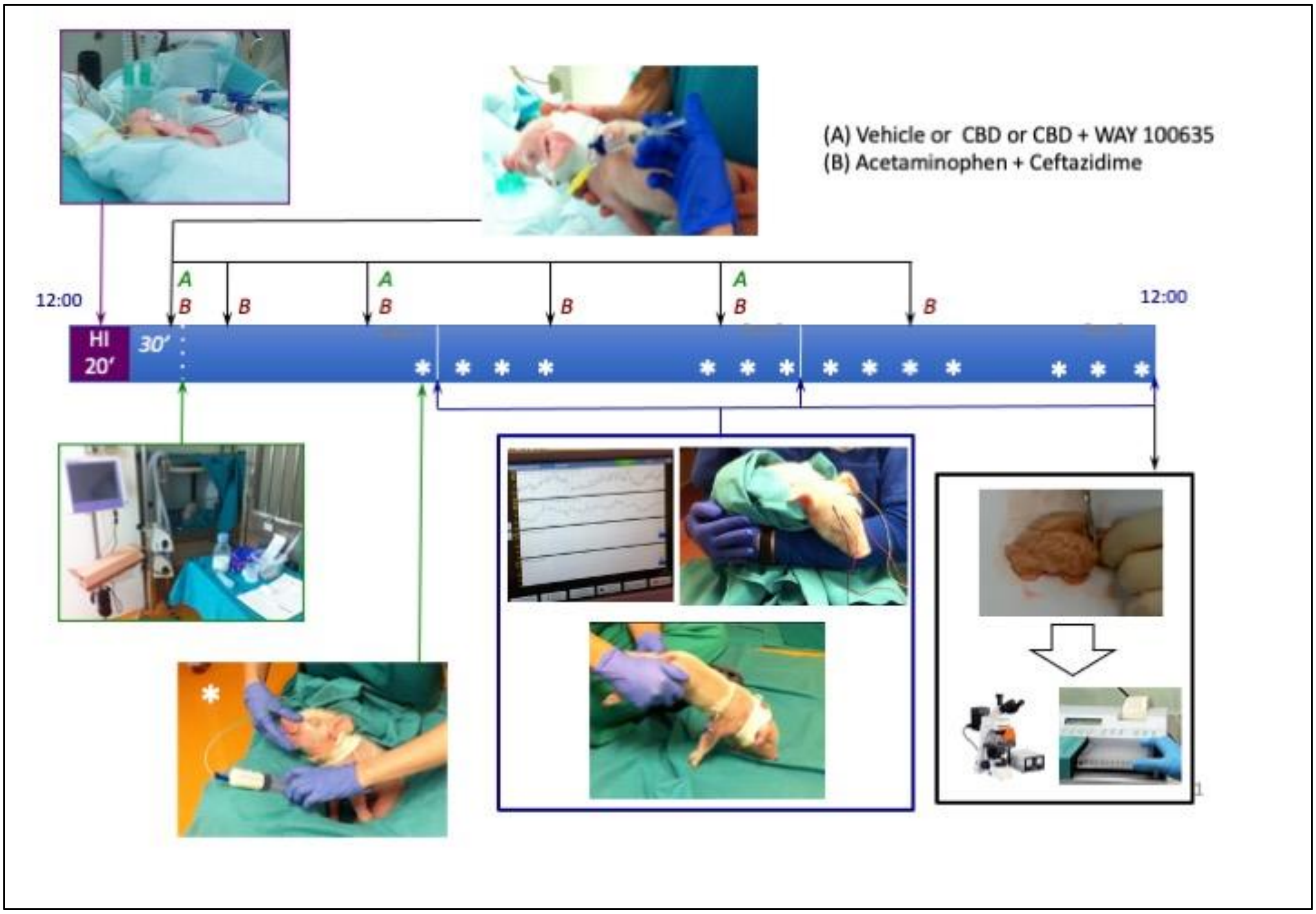

Fig.1S. Scheme of the experimental protocol. After the 20 min-long hypoxic-ischemic (HI) insult, piglets were allowed to recover. Thirty min after the end of HI piglets received i.v. (A) vehicle, cannabidiol 1 mg/kg (CBD) or CBD and WAY 100635 1 mg/kg; drug administration was repeated 24 and 48 h after the HI episode. In addition, piglets received (B) ceftazidime 15 mg/kg i.v. every 12 h to prevent infections and acetaminophen 15 mg/kg i.v. every 8 h for pain control. After the HI insult piglets were housed in stainless steel cages and periodically fed with artificial pig formula (asterisk). Each morning from 8 to 10 am a neurobehavioral test was carried out in the room where its cage was placed. After the neurobehavioral assessment, piglets were wrapped up with a blanket and held by an examiner sat in front of the aEEG device, to assess cerebral activity over 10 min. Seventy-two hours after the HI insult piglets were sacrificed by KCl infusion Then the brains were harvested and sectioned. Brain slices from the left hemisphere were placed into 4% paraformaldehyde for histologic analysis whereas those from the right hemisphere were snap frozen in isopentane and stored at -80ºC for spectroscopy studies.
